# Compositionally distinct nuclear pore complexes of functionally distinct dimorphic nuclei in the ciliate *Tetrahymena*

**DOI:** 10.1242/jcs.199398

**Published:** 2017-05-15

**Authors:** Masaaki Iwamoto, Hiroko Osakada, Chie Mori, Yasuhiro Fukuda, Koji Nagao, Chikashi Obuse, Yasushi Hiraoka, Tokuko Haraguchi

**Affiliations:** 1 Advanced ICT Research Institute, National Institute of Information and Communications Technology (NICT), Kobe 651-2492, Japan; 2 Graduate School of Agriculture, Tohoku University, Osaki, Miyagi 989-6711, Japan; 3 Graduate School of Life Science, Hokkaido University, Sapporo 001-0021, Japan; 4 Graduate School of Frontier Biosciences, Osaka University, Suita 565-0871, Japan; 5 Graduate School of Science, Osaka University, Toyonaka 560-0043, Japan

**Keywords:** FG-Nup, Nuclear dimorphism, Nuclear envelope, Nucleoporin, Y-complex

## Abstract

The nuclear pore complex (NPC), a gateway for nucleocytoplasmic trafficking, is composed of ∼30 different proteins called nucleoporins. It remains unknown whether the NPCs within a species are homogeneous or vary depending on the cell type or physiological condition. Here, we present evidence for compositionally distinct NPCs that form within a single cell in a binucleated ciliate. In *Tetrahymena thermophila*, each cell contains both a transcriptionally active macronucleus (MAC) and a germline micronucleus (MIC). By combining *in silico* analysis, mass spectrometry analysis for immuno-isolated proteins and subcellular localization analysis of GFP-fused proteins, we identified numerous novel components of MAC and MIC NPCs. Core members of the Nup107–Nup160 scaffold complex were enriched in MIC NPCs. Strikingly, two paralogs of Nup214 and of Nup153 localized exclusively to either the MAC or MIC NPCs. Furthermore, the transmembrane components Pom121 and Pom82 localize exclusively to MAC and MIC NPCs, respectively. Our results argue that functional nuclear dimorphism in ciliates is likely to depend on the compositional and structural specificity of NPCs.

## Abstract (precis)

**Summary:** There are compositional and structural differences in the nuclear pore complexes present in the functionally differentiated macronucleus and micronucleus within the single cytoplasm of ciliated protozoa.

## INTRODUCTION

**Fig. 1.**
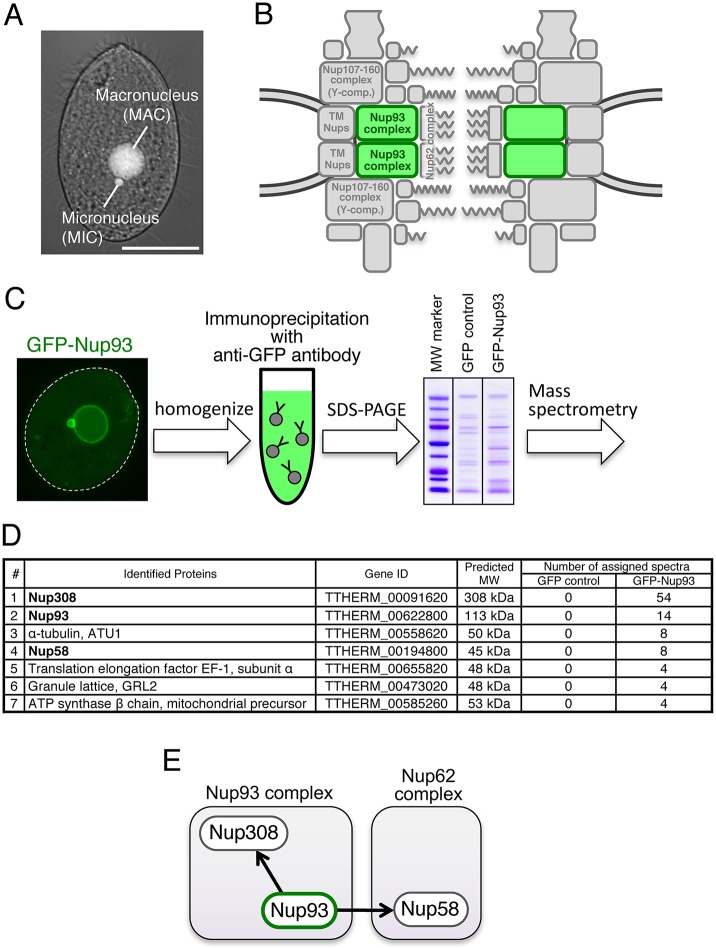
**Immunoprecipitation and mass spectrometry analysis to identify Nup93 complex members.** (A) A *T. thermophila* cell fixed with methanol and stained with DAPI to visualize the MAC and MIC. Scale bar: 20 μm. (B) The position of the Nup93 complex within the NPC architecture. See also Fig. S1. (C) Simplified procedure of immunoprecipitation and mass spectrometry for GFP–*Tt*Nup93-expressing cells used for immunoprecipitation. (D) Mass spectrometric identification of the proteins co-precipitated with GFP–*Tt*Nup93. The top seven proteins are listed among other identified proteins (further results are given in Table S2). (E) Physical interaction map of Nup93 based on the mass spectrometry results. MW, molecular mass.

Ciliated protozoa maintain two distinct nuclei within the same cytoplasm: a somatic macronucleus (MAC) and a germline micronucleus (MIC) (Fig. 1A) (Eisen et al., 2006; Orias et al., 2011; Karrer, 2012). The polyploid MAC is transcriptionally active, and its acentromeric chromosomes segregate during cell division by a spindle-independent amitotic process. In contrast, the diploid MIC has transcriptionally inert, centromeric chromosomes that segregate by canonical mitosis. In *Tetrahymena thermophila*, DNA replication in the MIC and MAC occurs during non-overlapping periods in the cell cycle. Thus, nuclear dimorphism in ciliates involves non-equivalent regulation of multiple activities in two distinct nuclei (Orias, 2000; Goldfarb and Gorovsky, 2009). This is likely to require targeted transport of components to the MIC versus MAC, for which differences in the NPCs may be important determinants.

Previously, we analyzed 13 *Tetrahymena* nucleoporins (Nups), and discovered that four paralogs of Nup98 were differentially localized to the MAC and MIC (Iwamoto et al., 2009). The MAC- and MIC-specific Nup98s are characterized by Gly-Leu-Phe-Gly (GLFG) and Asn-Ile-Phe-Asn (NIFN) repeats, respectively, and this difference is important for the nucleus-specific import of linker histones (Iwamoto et al., 2009). The full extent of the compositional differentiation of MAC and MIC NPCs could not, however, be assessed, since only a small subset of the expected NPC components were detected.

NPCs have been studied in eukaryotes including rat (Cronshaw et al., 2002), *Saccharomyces cerevisiae* (Rout et al., 2000), *Aspergillus nidulans* (Osmani et al., 2006), *Schizosaccharomyces pombe* (Asakawa et al., 2014), *Arabidopsis thaliana* (Tamura et al., 2010) and *Trypanosoma brucei* (Degrasse et al., 2009; Obado et al., 2016) (Table S1). The NPC structure has an 8-fold rotational symmetry, and is made up of roughly 30 known Nups organized into subcomplexes (Alber et al., 2007; Bui et al., 2013) (Fig. S1). The Nup93 complex [Nic96 in *S. cerevisiae*; hereafter orthologs in *S. cerevisiae* (*Sc*) are given in brackets at first mention where they have different names] in mammalian cells forms a stable scaffold composed of Nup93, Nup205 (*Sc*Nup192), Nup188, Nup155 (*Sc*Nup170 or *Sc*Nup157) and Nup53 (also known as Nup35 or MP-44; *Sc*Nup53 or *Sc*Nup59) (Grandi et al., 1997; Hawryluk-Gara et al., 2005; Amlacher et al., 2011). A second stable scaffold in mammals, the Nup107–Nup160 complex (called the Y-complex or Nup84 complex in *S. cerevisiae*) is composed of conserved subunits Nup107 (*Sc*Nup84), Nup160 (*Sc*Nup120), Nup133 (*Sc*Nup133), Nup96 (*Sc*Nup145C), Nup85 (*Sc*Nup85), Seh1 and Sec13, together with species-specific subunits (Siniossoglou et al., 1996; Lutzmann et al., 2002; Loiodice et al., 2004). Peripheral to the scaffolds are Phe-Gly (FG) repeat-bearing Nups, whose disordered FG-repeat regions constitute the central channel, with FG repeats interacting with nuclear transport receptors (Terry and Wente, 2009). Three transmembrane (TM) Nups anchoring the NPC to the mammalian nuclear membrane are NDC1, gp210 (also known as NUP210) and POM121 (Greber et al., 1990; Hallberg et al., 1993; Stavru et al., 2006) [*Sc*Ndc1, *Sc*Pom152 and *Sc*Pom34, respectively (Winey et al., 1993; Wozniak et al., 1994; Miao et al., 2006)]. A distinct nucleoplasmic basket is formed with Tpr (*Sc*Mlp1 or *Sc*Mlp2) (Cordes et al., 1997; Strambio-de-Castillia et al., 1999).

Based on prior analysis, *T. thermophila* appeared to lack homologs of many widely conserved NPC components. These included scaffold Nups (mammalian Nup205, Nup188, Nup160, Nup133, Nup107, Nup85 and Nup53, among others) from the Nup93 and Y-complexes. Similarly, homologs of FG-Nups Nup214, Nup153, Nup62 and Nup58 were also not detected, and neither were TM Nups except for gp210. These NPC components may have evaded homology-based searches due to extensive sequence divergence, given the large evolutionary distance between ciliates and animals, fungi and plants.

To address these ambiguities and to better understand NPC differentiation in *T. thermophila*, we attempted a comprehensive identification of Nups. First, we analyzed proteins that were affinity captured with known Nups. Furthermore, we mined updated genome and protein databases for characteristic Nup sequences or conserved domains through *in silico* structure prediction techniques. The resulting expanded catalog of *Tetrahymena* Nups, combined with localization data, sheds new light on the extent to which NPC architecture can vary within a single species, and even within a single cytoplasm.

## RESULTS

### The Nup93 complex includes a unique Nup205 ortholog and a novel central channel FG-Nup

In mammalian cells, the Nup93 complex (Fig. 1B) is composed of Nup93, Nup205, Nup188, Nup155 and Nup53 (Fig. S1) (Grandi et al., 1997; Hawryluk-Gara et al., 2005). In *T. thermophila*, we previously identified homologs for Nup93 (*Tt*Nup93; Gene Model identifier TTHERM_00622800) and Nup155 (*Tt*Nup155; TTHERM_00760460), and found both of them distributed to both MAC and MIC NPCs (Iwamoto et al., 2009). To identify other Nup93 complex components, we used mass spectrometry to analyze anti-GFP immunoprecipitates from *T. thermophila* expressing GFP–*Tt*Nup93 (Fig. 1C). All of the proteins listed in Table S2 as ‘hypothetical protein’ were examined by performing a Blast search for similarities to known Nups of other species. In addition, all of the ‘hypothetical proteins’ were examined through expression profile analysis in the *Tetrahymena* Functional Genomics Database (TetraFGD) website (http://tfgd.ihb.ac.cn/) [for details see the ‘Microarray’ page of the TetraFGD; http://tfgd.ihb.ac.cn/tool/exp (Miao et al., 2009) and also see Materials and Methods]. When either the Blast search or the expression profile analysis (details described below) found similarities to any known Nups, we examined its subcellular localization in *T. thermophila* by ectopically expressing GFP-fused proteins*.* By means of these analyses, we found Nup308 (TTHERM_00091620) and the novel protein TTHERM_00194800 (*Tt*Nup58; Nup58 in Fig. 1D and Table S2).

**Fig. 2.**
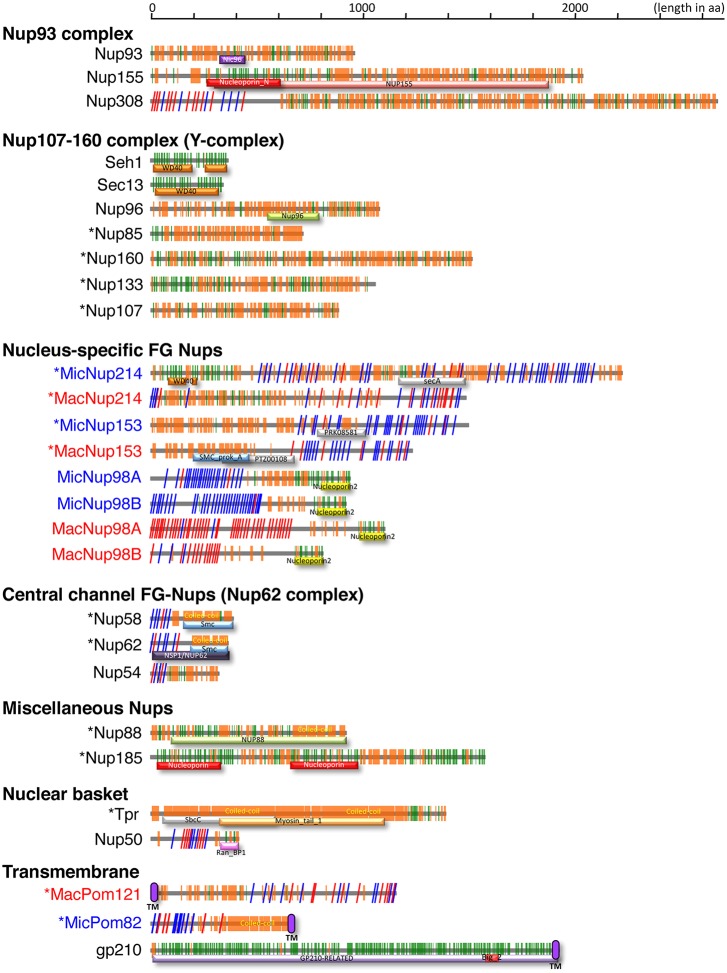
**Distributions of secondary structures and conserved domains in *Tetrahymena* nucleoporins.** Each Nup is shown as the protein name on the left. Blue, red and black letters denote MIC-specific, MAC-specific and shared components, respectively. Asterisks indicate Nups that are newly identified in this study. The colored components in the illustration are as follows: orange boxes/bars, α-helix; green boxes/bars, β-strand; red slanting lines, FG repeats; blue slanting lines, FX repeats (X means any residue, but the majority are N and Q); purple ellipses, predicted TM domain. Conserved domains are indicated by differently colored bars with standard domain names.

Nup308, a protein of 2675 amino acid residues, was previously identified as a *Tetrahymena*-specific Nup, but it was not assigned to a subcomplex (Iwamoto et al., 2009). Based on PSIPRED analysis, Nup308 is composed of GLFG repeats forming an N-terminal disordered structure (residues 1–570), followed by a large C-terminal α-helix-rich region (residues 571–2675) (Fig. 2). To identify potential Nup308 counterparts, we looked for Nups in other species with similar distributions of secondary structures. Interestingly, a large α-solenoid domain is a predicted feature of both Nup205 and Nup188, conserved core members of the Nup93 complex (Kosova et al., 1999; Andersen et al., 2013), although these proteins do not have FG repeats.

To investigate whether this structural similarity between *Tetrahymena* Nup308 and Nup205 and Nup188 homologs in other species reflected shared evolutionary origins, we performed a phylogenetic analysis. Nup308 formed a clade with Nup205 orthologs, supported by a bootstrap probability of 72%, but not with Nup188 orthologs (Fig. S2). Nup188 appears to be absent in *Tetrahymena*, since we failed to find any candidates in either the database or in our mass spectrometry data. Taken together, our results strongly suggest that Nup308 belongs to the Nup93 complex and is orthologous to human Nup205, but has acquired an unusual GLFG repeat domain. Consistent with this assignment, GFP–Nup308 localized similarly to GFP–*Tt*Nup93 in the cell, being equally distributed between MAC and MIC NPCs (Iwamoto et al., 2009).

The second Nup candidate identified in *Tt*Nup93 pulldowns was TTHERM_00194800. This small protein (deduced molecular mass of 45 kDa) is composed of an N-terminal FG-repeat region and a C-terminal coiled-coil region (Fig. 2), which are characteristics of central channel FG-Nups that are tethered by Nup93 (Chug et al., 2015). The secondary structure characteristics of the novel *Tetrahymena* Nup are highly similar to those of Nup62 and Nup58, central channel proteins in yeast and vertebrates that interact with Nup93 (Grandi et al., 1993, 1997). Because another protein was identified as an Nup62 ortholog (described below), this protein is the likely the *Tetrahymena* ortholog of Nup58; therefore, we named it *Tt*Nup58 (Nup58 in Fig. 1D,E).

### Newly identified members of the Y-complex are likely homologs of conserved Nups

**Fig. 3.**
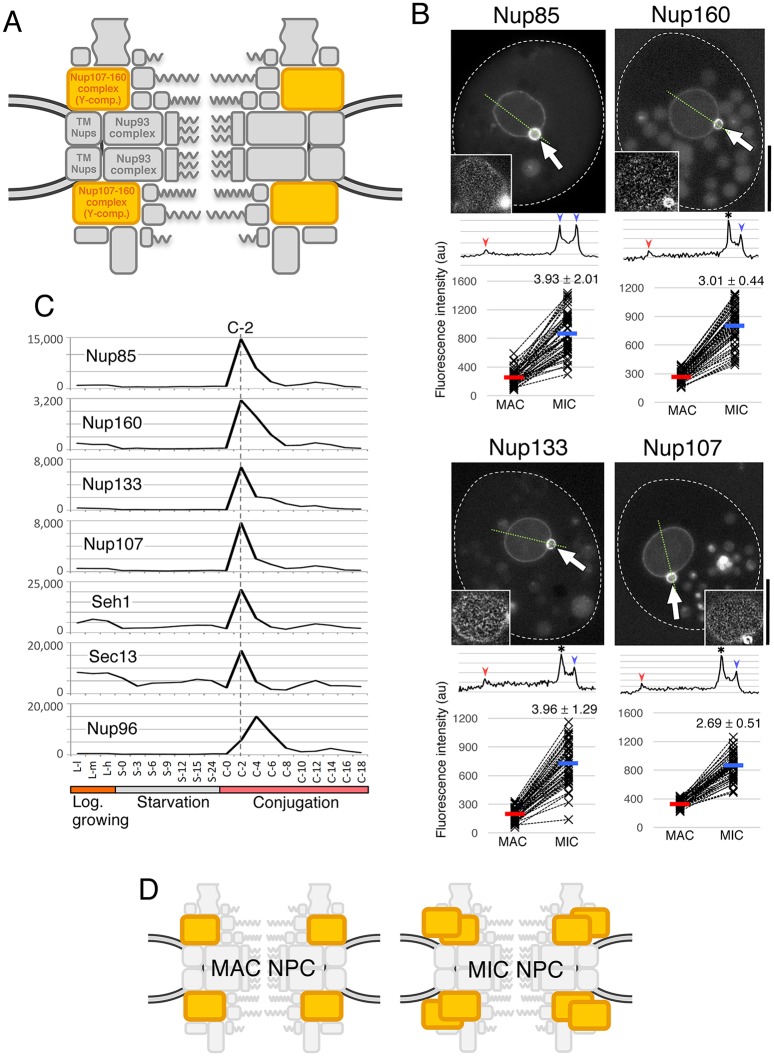
**Y-complex components localize to both nuclei but are biased to MICs.** (A) The position of the Y-complex (orange) within the NPC architecture. (B) Fluorescent micrographs of GFP-tagged Nups ectopically expressed in *Tetrahymena* cells. White broken lines represent the borders of cells. The inset in each panel shows a deconvoluted image focused on the MAC surface. Arrows indicate the position of the MIC. Scale bars: 20 μm. A line profile of fluorescence intensity along the thin green broken line is presented under each image panel. Blue and red arrowheads indicate the points corresponding to MIC and MAC envelopes, respectively. An asterisk marks the point at which the borders of the two nuclei overlap, and where the intensity is measured as the sum of both NEs. Below the line profile, the fluorescence intensities of MAC and MIC NEs from 50 cells are plotted. The vertical axis of the graph is shown in arbitrary units. Broken lines connect the plots of MAC and MIC within the same cell. Average values are presented by red and blue bars for the MAC and MIC, respectively. The numbers upon the MIC plots indicate fold increase (±s.d.) of fluorescence in MIC from MAC. All differences are significant (*P*<10^−20^ by Student's *t*-test). (C) Expression profiles of the Y-complex members extracted from the TetraFGD (http://tfgd.ihb.ac.cn/). Plots are the average of two values presented in the database. The horizontal axis represents successive stages of culture growth and therefore different physiological conditions. For the logarithmic growth stage, L-l, L-m, and L-h represent low, medium, and high cell concentrations, respectively. For starvation and conjugation stages, numbers represent hours after the transfer of the cells to each condition. The vertical axis represents relative values of mRNA expression. For details, visit the database website. (D) A simple representation of the deduced composition of MAC and MIC NPCs with different numbers of Y-complexes.

The Y-complex in vertebrates (Fig. 3A) contains ten distinct proteins (Orjalo et al., 2006; Mishra et al., 2010), of which three had identified homologs in *T. thermophila* (*Tt*Seh1, *Tt*Sec13 and *Tt*Nup96) (Iwamoto et al., 2009). To investigate whether the remaining seven are present in *Tetrahymena* but had been overlooked due to sequence divergence, we carried out mass spectrometric analysis of anti-GFP immunoprecipitates from cells expressing the known Y-complex GFP-tagged Nups described below.

First, in precipitates of GFP–*Tt*Seh1, we identified an 86 kDa protein orthologous to Nup85 (Table S3) with a short stretch of four predicted β-strand blades at the N-terminus followed by an α-solenoid domain (Fig. 2). That architecture is typical of Nup85 orthologs that are Y-complex components in other organisms (Brohawn et al., 2008). We therefore tentatively named the *T. thermophila* protein *Tt*Nup85. GFP–*Tt*Nup85 localized to NPCs in both the MAC and MIC (Fig 3B; Fig. S3A).

We then immunoprecipitated GFP–*Tt*Nup85, and identified two novel candidate Y-complex core members. Both proteins are comprised a β-strand-rich N-terminal half and an α-helical-rich C-terminal half. This domain architecture is characteristic of the Y-complex components Nup160 and Nup133 (Berke et al., 2004; Devos et al., 2004), and we tentatively named the *Tetrahymena* proteins *Tt*Nup160 and *Tt*Nup133 (Fig. 2; Table S4). GFP–*Tt*Nup160 and GFP–*Tt*Nup133 localized to NPCs in both nuclei, like other Y-complex components (Fig. 3B; Fig. S3A).

Another conserved Y-complex component is Nup107, which interacts with Nup96. To search for the *Tetrahymena* homolog we used GFP–*Tt*Nup96 as bait and identified a 109 kDa protein (Table S5) that is rich in predicted α-helices, like human Nup107 (Fig. 2). The protein, tentatively named *Tt*Nup107, localized as a GFP-tagged construct to NPCs of both nuclei (Fig 3B; Fig. S3A).

The genes encoding all members of the Y-complex except for Nup96 are co-expressed and exhibit sharp expression peaks at 2 h (denoted C-2) after two cell strains with different mating-types were mixed for conjugation [for details see the ‘Microarray’ page of the TetraFGD at http://tfgd.ihb.ac.cn/tool/exp (Miao et al., 2009)] (Fig. 3C). In contrast, *Tt*Nup96 exhibits an expression peak at 4 h (C-4). This difference in the timing of expression between *Tt*Nup96 and the other Y-complex components may be related to a unique aspect of *Tt*Nup96 gene structure: *Tt*Nup96 is expressed as part of a single transcription unit together with MicNup98B, under the promoter of the MicNup98B gene (Iwamoto et al., 2009).

Three other components of the human Y-complex were not detected in our studies: Nup43, Nup37 and ELYS (also known as AHCTF1). These components may be species specific (Bilokapic and Schwartz, 2012; Rothballer and Kutay, 2012) and genuinely absent from *Tetrahymena*. They are also absent from *S. cerevisiae* (Alber et al., 2007) (see Table S1), supporting this idea.

### Y-complex components show biased localization to the MIC

As previously reported, GFP-tagged Nup93 complex members and some of the central channel Nups (*Tt*Nup93, *Tt*Nup308 and *Tt*Nup54) were distributed equally between MAC and MIC NPCs, judging by fluorescence intensities (Iwamoto et al., 2009). In striking contrast, all Y-complex components identified so far exhibit distinctively biased localization to the MIC nuclear envelope (NE) compared to the MAC NE (Fig. 3B). Fluorescence intensities in the MIC were 2.69–3.96 times higher than those in the MAC (Fig. 3B). This biased localization of Y-complex components might have been caused by overexpression of the components due to the ectopic expression the GFP-tagged proteins in addition to the expression of endogenously untagged ones. To address this issue, we examined the localization of Nup160–GFP, Nup133–GFP and Seh1–mCherry expressed from endogenous loci under the control of their native promoters, and therefore expressed at physiological levels. All three proteins showed biased localization, as found for the overexpressed GFP-tagged proteins (compare the images in Fig. 3B and Fig. S3B), suggesting that the biased localization is not caused by overexpression of the tagged proteins. Because the NPC density is similar in the MAC and MIC (Fig. S1 in Iwamoto et al., 2009), the relative concentration of Y-complex components in the MIC NE suggests that the Y-complex is present at a higher copy number per NPC in the MIC compared to in the MAC (Fig. 3D).

### Newly detected FG-Nups include nucleus-specific and common components

**Fig. 4.**
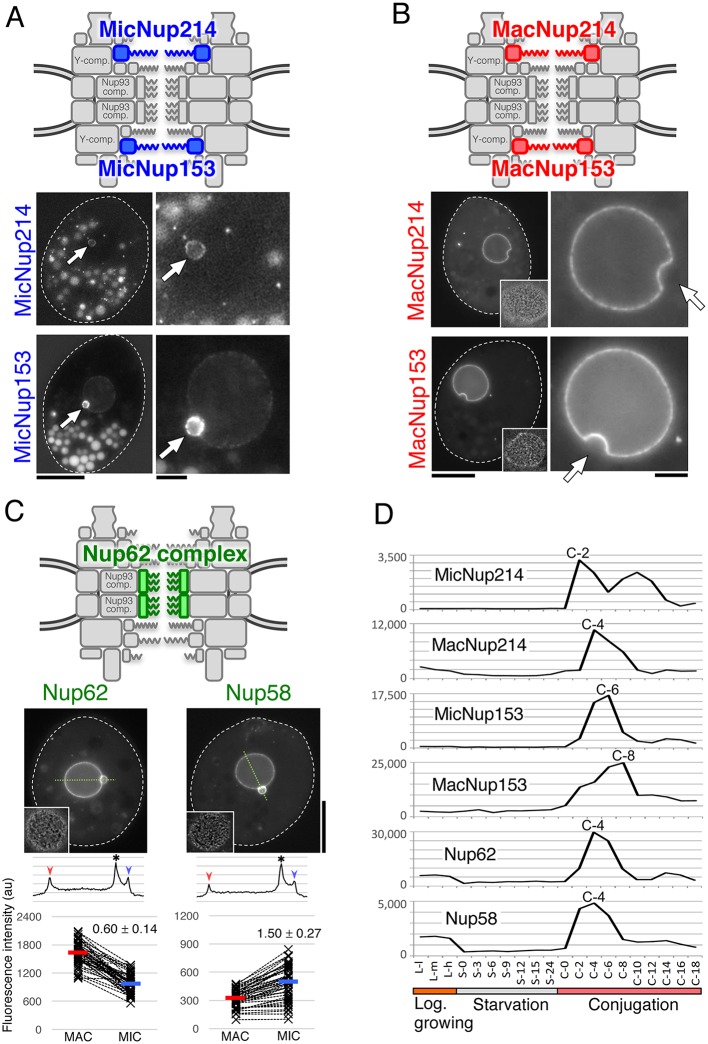
**Newly identified FG-Nups of *Tetrahymena.*** (A) MIC-specific paralogs of Nup214 and Nup153. The upper figure indicates the predicted positions of these Nups within the MIC NPC. Fluorescence micrographs show the subcellular localization of fluorescent protein-tagged Nups; MicNup214 and MicNup153 were endogenously tagged with GFP and mNeon at the C-termini of their ORFs, respectively. Arrows indicate the position of the MIC. Other fluorescent bodies dispersed in the cytoplasm are phagosomes taking in materials derived from the culture medium. (B) MAC-specific paralogs of Nup214 and Nup153. The upper figure indicates the predicted positions of these Nups within the MAC NPC. Fluorescence micrographs show the subcellular localizations of ectopically expressed GFP-tagged Nups. The left panels show a whole cell, and each nuclear region is enlarged in the right panels. White broken lines represent the borders of cells. Insets in the left panels show deconvoluted images focused on the MAC surface. Arrows indicate the position of MICs. (C) *Tt*Nup62 and *Tt*Nup58 localized to both nuclei. The upper illustration indicates the predicted position of these Nups, which are members of the Nup62 complex. Fluorescent micrographs show the subcellular localizations of ectopically expressed GFP–*Tt*Nup62 and *Tt*Nup58–GFP. Line profiles and plots of fluorescence intensity are shown under each image panel in the same manner as in Fig. 3B. Both differences are significant (*P*<10^−16^ by Student's *t*-test). (D) Expression profiles of FG-Nups, as in Fig. 3C. Scale bars: 20 μm (A,B, left panels; C); 5 μm (A,B, right panels).

FG-Nups were originally characterized as nucleoporins with domains containing extensive repeats of phenylalanine-glycine (FG) that function in nucleocytoplasmic transport. More recently, we reported a remarkable difference in MAC and MIC NPCs regarding the repeat signature present in four Nup98 paralogs. The repeat signature of MacNup98A and MacNup98B is mostly GLFG, while that of MicNup98A and MicNup98B is mostly NIFN (Fig. 2) (Iwamoto et al., 2009, 2010, 2015). We have now taken advantage of the recently improved annotation of the *Tetrahymena* Genome Database Wiki (http://ciliate.org/index.php/home/welcome), to search for sequences bearing repeats that are similar to those of FG-Nups in other species. We found five candidate FG-Nups. Based on the molecular mass and the positions of predicted α-helices, β-strands and FG-repeat regions, we designated four of these proteins as MicNup214 (TTHERM_00992810), MacNup214 (TTHERM_00755929), MicNup153 (TTHERM_00647510) and MacNup153 (TTHERM_00379010); GFP fusions of MicNup214 and MacNup214 were exclusively localized to the MIC and MAC, respectively (Fig. 4A,B). Fluorescent protein (GFP or mNeon) fusions of MicNup153 were primarily localized to the MIC, with less localizing to the MAC, in most growing cells (Fig. 4A), although these fusion proteins were exclusively localized to the MIC in some cells (Fig. S3C). GFP fusions of MacNup153 were exclusively localized to the MAC (Fig. 4B). The localization of the fifth candidate FG-Nup (*Tt*Nup62; Nup62 in Fig. 4C), like the novel nucleoporin *Tt*Nup58 (Nup58 in Fig. 4C) identified as a central channel protein (discussed above), showed less-specific distribution on both MAC and MIC.

A striking feature of the Nup214 paralogs is that they contain the same nucleus-specific repeat motifs described earlier for *Tt*Nup98 paralogs. Like the MIC-specific Nup98 paralogs, MicNup214 contains NIFN repeats (the last N is usually Q in this protein), while MacNup214 contains FG repeats (Fig. 2). This difference may be an important determinant for selective protein transport to the MAC and MIC, as previously shown for *Tt*Nup98 s (Iwamoto et al., 2009). We note that MacNup214 lacks the β-strand-rich N-terminal region that is found in other Nup214 orthologs (Weirich et al., 2004; Napetschnig et al., 2007) (Fig. 2).

In contrast, MicNup153 and MacNup153 do not differ markedly from one another in their molecular features (Fig. 2). Because the N-terminus domain of human Nup153 is involved in its NPC localization (Enarson et al., 1998), we speculate that the N-terminal domains of MicNup153 and MacNup153 may also be involved in their nucleus-specific localization in *Tetrahymena*. Further study is required to elucidate their nucleus-specific localization.

While the expression of this set of FG-Nups is upregulated during conjugation (Fig. 4D), the MIC-specific components tend to be expressed 2 h earlier than MAC-specific ones. For example, MicNup214 expression peaks at 2 h in conjugation (C-2) versus MacNup214 at C-4; similarly, MicNup153 peaks at C-6 versus MacNup153 at C-8 (Fig. 4D). The earlier expression of MIC-specific components compared with MAC-specific ones may reflect a selective requirement for MIC-specific NPCs during early stages of conjugation, such as the crescent stage (Sugai and Hiwatashi, 1974). In contrast, the later expression of MAC-specific components probably reflects formation of the new MACs that occurs in the later stages of conjugation.

The fifth candidate FG-Nup identified by this screen was a 39 kDa protein (TTHERM_01122680). This protein is composed of an N-terminal FG-repeat region and a C-terminal coiled-coil region with the characteristics of central channel FG-Nups and is assigned as a nucleoporin NSP1/NUP62 family protein (IPR026010) (Fig. 2). Consequently, this protein is the likely *Tetrahymena* ortholog of Nup62; therefore, we named it *Tt*Nup62. The GFP-tagged protein was distributed to both nuclei (Nup62 in Fig. 4C), similarly to the central channel Nups *Tt*Nup58 (Figs 1E and 4C) and *Tt*Nup54 (Iwamoto et al., 2009), although *Tt*Nup62 was slightly enriched in the MAC NE, whereas *Tt*Nup58 was slightly enriched in the MIC NE. The expression profile of *Tt*Nup62 was similar to that of *Tt*Nup58, with an expression peak after 4 h of conjugation (C-4) (Fig. 4D).

*Tt*Nup62 has relatively few repeats in its FG motif compared with homologs such as human Nup62 and *S. cerevisiae* Nsp1 (Fig. 2), although it has several FX repeats (X=N, Q, A or T in the case of this protein). A feature unique to *Tetrahymena* is the presence of GLFG repeats in Nup308, an ortholog of Nup205. The Nup93 complex containing Nup205 anchors Nup62 (Vollmer and Antonin, 2014), and it is likely that the *Tetrahymena* Nup93 complex containing Nup308 anchors *Tt*Nup62. Thus, we hypothesize that the GLFG repeats present in Nup308 compensate for the low number of FG repeats of *Tt*Nup62 present in the central channel.

### Nup88, Nup185 and Tpr

**Fig. 5.**
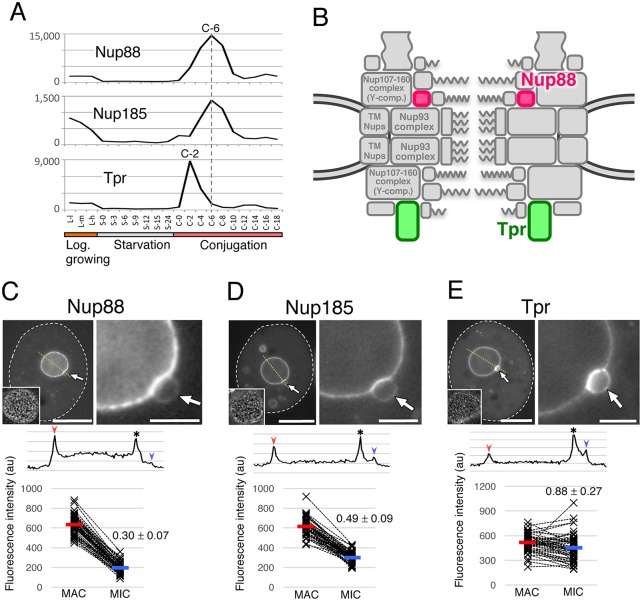
**Nuclear localization and expression profiles of Nup88, Nup185 and Tpr.** (A) Expression profiles of Nup88, Nup185 and Tpr. (B) The predicted positions of *Tt*Nup88 and *Tt*Tpr in the NPC. The position of Nup185 is unknown. (C) The subcellular localization of ectopically expressed GFP–*Tt*Nup88. The fluorescence intensity of the MIC NE is significantly lower than that of the MAC NE (*P*<10^−39^). (D) Subcellular localization of ectopically expressed GFP–Nup185. The fluorescence intensity of the MIC NE is significantly lower than that of the MAC NE (*P*<10^−30^). (E) Subcellular localization of ectopically expressed GFP–*Tt*Tpr. The fluorescence intensity of the MIC NE is slightly lower than that of the MAC NE (*P*=0.0024). The left panels in C–E show a whole cell, and its nuclear region is enlarged in the right panels. White broken lines represent the borders of cells. The inset in the left panels show the deconvoluted image focused on the MAC surface. Arrows indicate the position of the MICs. A line profile and plots of fluorescence intensity are shown under each image panel, as in Fig. 3B. All *P*-values were calculated with a Student's *t*-test. Scale bars: 20 μm (C–E, left panels); 5 μm (C–E, right panels).

We used a variety of strategies to identify additional Nups. Homology searches against InterPro (http://www.ebi.ac.uk/interpro/) revealed a gene (TTHERM_00455610) with a conserved Nup88 domain ‘*Tt*Nup88 (PTHR13257:SF0)’ (Fig. 2) and an expression profile similar to those of some other *Tetrahymena* Nups (Fig. 5A). Localization of a GFP fusion to NPCs was highly biased, albeit not exclusive, to the MAC (Fig. 5C). We therefore named this protein *Tt*Nup88, and it is known that it localizes to the cytoplasmic side of the NPC in other species (Fig. 5B). As Nup88 in other species is known to interact with Nup214 and Nup98 (Fornerod et al., 1997), *Tt*Nup88 may contribute to the nucleus-specific localization of Nup214 and Nup98 paralogs.

TTHERM_00755920 (encoding a 185 kDa protein), which lies adjacent to the open reading frame (ORF) of MacNup214, attracted our interest because its predicted molecular structure resembled those of large scaffold Nups such as Nup160, Nup155 and Nup133, and because its expression profile is similar to those of some other *Tetrahymena* Nups (Fig. 5A). A GFP fusion localized to NPCs, with a bias to the MAC (Fig. 5D). Based on its predicted molecular mass, we named this protein Nup185. Nup185 contains a conserved domain annotated as ‘Nucleoporin’ in the SUPERFAMILY database (SSF117289) (Fig. 2), which is generally found near the N-terminal regions of Nup155 and Nup133 homologs. The expression peak of Nup185 appeared at C-6 (Fig. 5A).

To assess the location of Nup185 within the NPC architecture, we identified interacting proteins by immunoprecipitating GFP–Nup185. One interacting protein was TTHERM_00268040, which bears predicted coiled-coil motifs throughout its entire sequence (Fig. 2) and is thus similar to the nuclear basket component Tpr (Fig. 5B). TTHERM_00268040 fused with GFP localized equivalently to MAC and MIC NPCs (Fig. 5E). This protein is a likely ortholog of human Tpr; therefore, we named it *Tt*Tpr. Nup185 did not interact with any members of the Y- or Nup93 complexes (Table S6).

### The TM Nups Pom121 and Pom82 show nucleus-specific localization

**Fig. 6.**
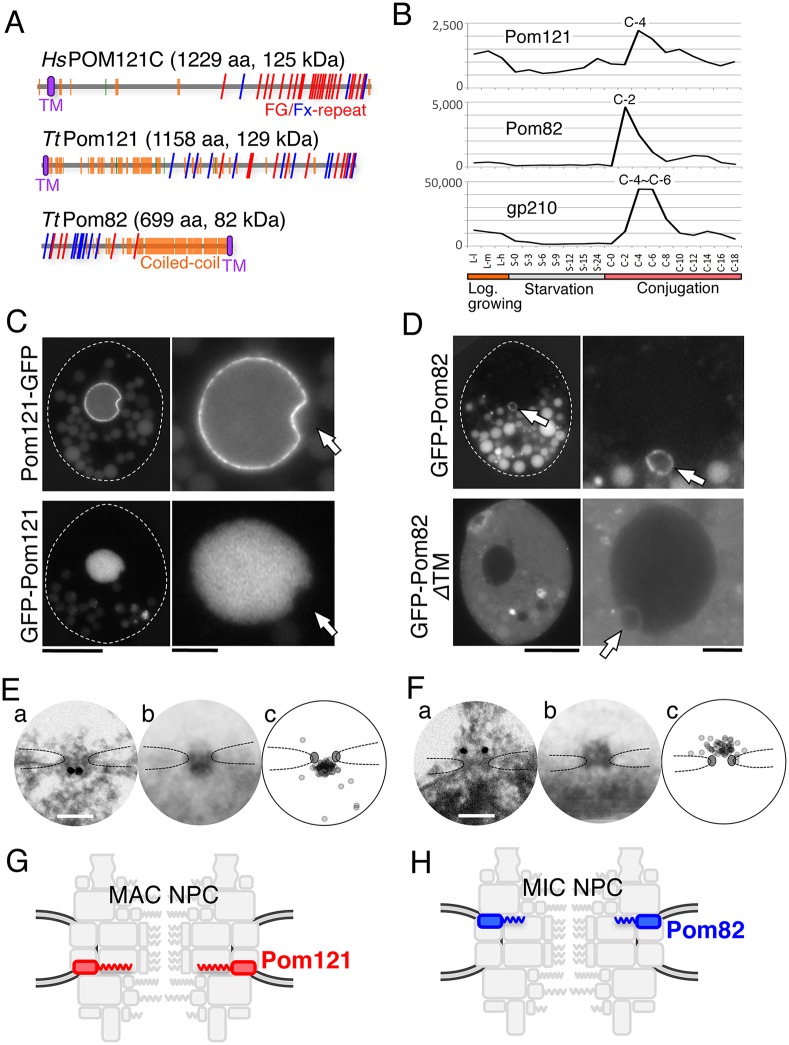
**Two novel pore membrane proteins show nuclear specificity.** (A) Illustration of molecular profiles. The frequency and positions of FG repeats are compared between *T. thermophila* Pom proteins and human POM121C (UniProt A8CG34). Red and blue slanting lines represent FG and FX (X means any amino acid residue, but the majority are N, Q and S) repeats, respectively. Orange and green boxes represent α-helices and β-strands, respectively. Purple ellipses represent predicted TM domains. (B) The expression profiles of nuclei-specific Pom proteins (MAC for Pom121 and MIC for Pom82) and shared *Tt*gp210, as in Fig. 3C. (C) Fluorescence micrographs of ectopically expressed GFP-tagged *Tt*Pom121. Left panels show whole cells, and the right panels show enlarged images of the nuclear regions. White broken lines represent the borders of cells. Arrows indicate the position of MICs. Bars indicate 20 μm for the left panels and 5 μm for the right panels. (D) Fluorescence micrographs as in C showing GFP-tagged Pom82 (full length, amino acids 1–699) and GFP–Pom82ΔTM (transmembrane domain-deletion mutant, amino acids 1–678), both ectopically expressed. Arrows indicate the position of the MICs. Other fluorescent bodies dispersed in the cytoplasm are phagosomes taking in materials derived from the culture medium. (E,F) iEM for Pom121–GFP localizing to the MAC NPC (E) and GFP–Pom82 localizing to the MIC NPC (F) as determined by using anti-GFP antibody. (a) Immuno-electron micrographs for a single NPC. Dark dots represent signals of gold particles. Scale bars: 100 nm. (b) Images present a projection image of 20 immuno-electron micrographs of NPCs decorated with gold particles. (c) The positions of individual gold particles in b are plotted. Broken lines trace nuclear envelope, and upper and lower sides are cytoplasm and nucleoplasm, respectively. (G) The position of *Tt*Pom121 within the MAC NPC architecture. (H) The position of *Tt*Pom82 within the MIC NPC architecture.

Some but not all of the TM Nups are conserved between vertebrates and yeasts: the former have POM121, gp210 and NDC1 (Cronshaw et al., 2002; Stavru et al., 2006), while the latter have Pom34, Pom152 and Ndc1 (Rout et al., 2000; Asakawa et al., 2014). The only reported TM Nup in *T. thermophila* is gp210 (Iwamoto et al., 2009). Because all *Tetrahymena* Nups identified so far have a similar expression pattern in which a large expression peak appears during early conjugation stage (Figs 3C, 4C and 5A), we used expression profiling and TM domain search to identify possible TM Nups in the updated TetraFGD and the TMHMM Server (http://www.cbs.dtu.dk/services/TMHMM-2.0/), respectively. By using this approach, we found two candidate TM Nups. Each has one TM domain and an FG-repeat region (‘*Tt*Pom121’ and ‘*Tt*Pom82’ in Fig. 6A). Their expression profiles are shown in Fig. 6B.

One of the TM Nup candidates (TTHERM_00312730; *Tt*Pom121) has an N-terminal TM domain and C-terminal FG repeats (Fig. 6A, middle) with a deduced molecular mass of 129 kDa. These attributes are very similar to those of vertebrate POM121 (compare top and middle parts of Fig. 6A) (Rothballer and Kutay, 2012). *Tt*Pom121 fused with GFP at its C-terminus (*Tt*Pom121–GFP) localized specifically to MAC NPCs (Fig. 6C, upper). Consequently, this protein is the likely the *Tetrahymena* ortholog to human POM121; therefore, we named it *Tt*Pom121.

Notably, when GFP was fused with the N-terminus of *Tt*Pom121 at a region close to the TM domain (GFP–*Tt*Pom121), the tagged protein localized in the MAC nucleoplasm, but not in MAC NPCs or the MIC nucleoplasm (Fig. 6C, lower panels). This result suggests that *Tt*Pom121 bears a MAC-specific nuclear localization signal (NLS) in its N-terminal region. Similarly, POM121 homologs in vertebrates have NLS sequences in the N-terminal region (Yavuz et al., 2010; Funakoshi et al., 2011).

In contrast, the other TM Nup candidate (TTHERM_00375160; *Tt*Pom82) localized exclusively to MIC NPCs (Fig. 6D, upper). This protein has predicted molecular features that have not been reported in Nups from any other organism: a TM domain near the C-terminus, a central coiled-coil and N-terminal FG repeats (Fig. 6A, bottom). We named this protein *Tt*Pom82 according to its predicted molecular mass (82 kDa). A construct lacking the TM domain showed diffuse cytoplasmic localization (Fig. 6D, lower panels), suggesting that MIC NPC-specific localization of *Tt*Pom82 does not depend on the MIC-specific nuclear transport of *Tt*Pom82. This result suggests that *Tt*Pom121 and *Tt*Pom82 use different mechanisms to target to the MAC and MIC NPCs.

Next, we performed immuno-electron microscopy (iEM) for the Pom proteins using anti-GFP antibody in order to determine their sub-NPC localization. Intriguingly, their sub-NPC localizations were opposite; Pom121 was exclusively localized to the nuclear side of the MAC NPC (Fig. 6E), whereas Pom82 was exclusively localized to the cytoplasmic side of the MIC NPC (Fig. 6F).

**Fig. 7.**
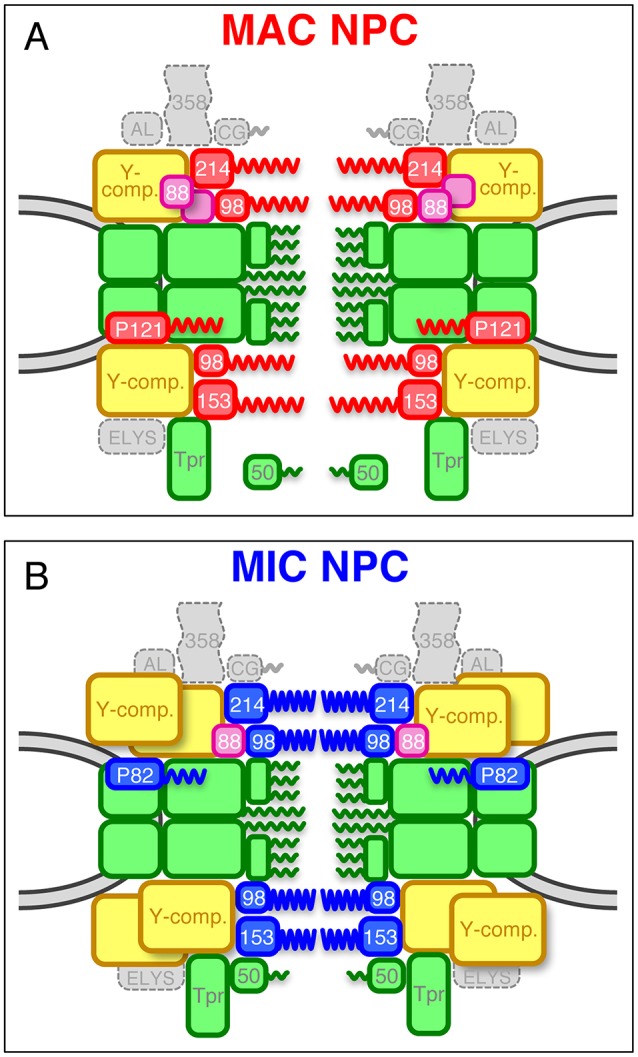
**Schematic models of MAC and MIC NPCs.** (A) Deduced composition of the MAC NPC. (B) Deduced composition of the MIC NPC. Boxes colored in red and blue represent MAC-specific and MIC-specific components, respectively [P121, Pom121; P82, Pom82; 98, Nup98 paralogs; 214, Nup214; 153, Nup153]. Green boxes represent shared components including the nuclear basket structure Tpr and its associated Nup50 (50). *Tt*Nup50 is distributed mostly in the nucleoplasm in MACs, whereas it localizes to the NPC in MICs (Malone et al., 2008; Iwamoto et al., 2009). Yellow boxes are MIC-biased Y-complexes, and purple boxes are MAC-biased *Tt*Nup88 (88). The number of duplications of yellow and purple boxes does not reflect the actual quantity of those components *in vivo*. Homologs of Nup358 (358), hCG1 (CG), Aladin (AL), and ELYS constituting the cytoplasmic structure, were not found in *T. thermophila*.

Given the difference in molecular features, their behaviors when the TM domain function was disrupted, and their sub-NPC localizations, Pom121 and Pom82 are unlikely to be functional homologs of each other. Taken together, these findings lead to the conclusion that MAC and MIC NPCs contain distinct TM components (Fig. 6G,H). The protein components of MAC and MIC NPCs are summarized in Fig. 7.

One TM Nup, found in both fungi and animals but missing from our *Tetrahymena* catalog, is Ndc1. We identified a potential Ndc1 homolog in TTHERM_00572170, a protein with six predicted TM domains that is co-transcribed with other Nups (see http://tfgd.ihb.ac.cn/search/detail/gene/TTHERM_00572170). However, neither N- nor C-terminal GFP fusions of this protein localized to NPCs (Fig. S3D). Therefore, *Tetrahymena* NPCs may lack Ndc1. Similarly, Ndc1 has not been detected in *Trypanosoma* NPCs (Obado et al., 2016).

### The permeability of the nuclear pore differs between MAC and MIC

To better understand the functional consequences of structural differences between MAC and MIC NPCs, we examined the relative pore exclusion sizes by asking whether probes of different sizes could gain access to each nucleoplasm. GFP (∼28 kDa) was excluded only from MICs, whereas GFP–GST (more than 100 kDa owing to its oligomerization) was excluded from both MACs and MICs (Fig. S4A). In addition, FITC–dextran of 40 kDa could enter MACs, whereas 70-kDa FITC–dextran was completely excluded (Fig. S4B). These results indicate that MAC pores exclude molecules greater than ∼50 kDa, which is similar to the permeability size limit of nuclear pores in other species (Paine et al., 1975; Gorlich and Mattaj, 1996; Keminer and Peters, 1999). On the other hand, MIC pores impose a much smaller exclusion size, and exclude molecules of even 10–20 kDa (Fig. S4B). This difference in exclusion size may be due to differences between the protein composition and structural arrangement of NPCs of these dimorphic nuclei.

## DISCUSSION

We have now identified 28 nucleoporins in the ciliate *T. thermophila*: 15 Nups reported here, and 13 in our previous study (Iwamoto et al., 2009). This total comprises 24 different Nups for the MAC and MIC: this number includes 18 Nups that are localized in both nuclei, four Nups with nucleus-specific homologs (Nup214, Nup153, Nup98A, and Nup98B), and *Tt*Pom82 and *Tt*Pom121. This total is somewhat smaller than the roughly 30 Nups known in other eukaryotes, e.g. 34 in human and in *Drosophila melanogaster*, 27 in *Caenorhabditis elegans*, 33 in *S. pombe* and 35 in *S. cerevisiae* (Rothballer and Kutay, 2012; Asakawa et al., 2014). The deficit in *T. thermophila* Nups is due to the absence of homologs for Nup358, GLE1, human CG1 (also known as NUPL2; *Sc*Nup42), Nup43, Nup37, centrin-2, Nup53, TMEM33, ELYS and Aladin. Similarly, the protist *Trypanosoma brucei* is missing homologs of Nup358, GLE1, human CG1, Nup37, centrin-2, TMEM33 and ELYS, and 25 Nups in total have been identified by interactome analysis (DeGrasse et al., 2009; Obado et al., 2016). One conserved Nup identified in *Trypanosoma* but not *Tetrahymena* is Nup53 (*Tb*Nup65; Genbank XP_822630.1) (Obado et al., 2016). This raises the question of whether a *T. thermophila* Nup53 homolog eluded our search due to sequence or structural divergence. Alternatively, *T. thermophila* may have lost a Nup that is not essential for viability.

### A role for nucleus-specific Nups

We previously reported that the GLFG-repeat and NIFN-repeat domains in MacNup98A and B, and MicNup98A and B, respectively, are involved in the nucleus-specific transport of linker histones (histone H1 and MLH, respectively), arguing that these nucleus-specific Nups are determinants of nucleus-specific transport (Iwamoto et al., 2009). Importantly, we can now expand this argument, since our expanded catalog shows that all NPC subunits that are nucleus-specific are FG-Nups (Nup214, Nup153, Nup98 and Pom proteins). Since the FG repeats interact with nuclear transport receptors such as importin-β family proteins (Allen et al., 2001; Isgro and Schulten, 2005; Liu and Stewart, 2005; Tetenbaum-Novatt et al., 2012), specificity for the MAC or MIC is likely to be determined in cooperation with importin-βs. This idea is also supported by the presence of nucleus-specific importin family proteins (Malone et al., 2008).

It is interesting to note that both MAC- and MIC-specific Nups contain atypical repeat motifs including the NIFN motif and also more subtle variations on the FG repeat (FN, FQ, FA, FS and so on) (Fig. 2). Because the NIFN-repeat domain of MicNup98A is known to function in blocking misdirected nuclear transport of MAC-specific linker histones (Iwamoto et al., 2009), the atypical FG repeats may similarly be involved in controlling nucleus-specific transport of particular proteins. However, importin-βs that preferentially interact with the NIFN repeat and their cargos have not been found, and thus the complete role of the NIFN-repeat motif in nucleus-specific transport remains to be elucidated.

### A role of biased Nups to build different NPC structures

The nucleus-specific Nups generate obvious structural differences between MAC and MIC NPCs. However, these different components have to be integrated into two NPC scaffold structures that are constructed of the same components. One way to make different structures from the same components is to incorporate different amounts of these components, leading to different structures that allow biased localization/assembly of nucleus-specific components. The localization of the Y-complex (Fig. 3B) and Nup88 (Fig. 5C) was highly biased to either MICs or MACs, respectively. Thus, these biased components may be critical for directing assembly of MAC- or MIC-type NPCs. Consistent with this idea, Nup98 homologs in vertebrates interact with the Y-complex components Nup96 (Hodel et al., 2002) and Nup88 (Griffis et al., 2003). This model raises the question of how structurally similar paralogs in *Tetrahymena* can differentially recruit nucleus-specific FG-Nups.

The copy number of the Y-complex within individual NPCs differs between the MAC and MIC (Fig. 3B,D), indicating that at least two NPC structures with different Y-complex stoichiometries can form in ciliates. This quantitative difference in Y-complex incorporation may be directed by membrane Nups. The nucleus-specific TM Nups Pom121 and Pom82 are currently strong candidates for initiating NPC assembly on the nuclear membrane. In vertebrates, Pom121 binds the Y-complex through a Nup160 homolog (Mitchell et al., 2010). In *Tetrahymena*, *Tt*Pom121 and *Tt*Pom82 may differentially affect Y-complex integration into MAC or MIC NPCs. This model can be extended to biased integration of Nup98 paralogs, since Pom121 has been shown to directly bind Nup98 proteins (Mitchell et al., 2010), supporting our idea that biased Nups and nucleus-specific Nup98 paralogs cooperate to build two distinct NPCs. In this model, the acquisition of specialized Pom proteins might have been one of the most crucial evolutionary events for generating nuclear dimorphism in ciliates. Taken overall, our study contributes to understanding the diversity of NPC architectures in eukaryotes, including potential functional and evolutionary aspects.

## MATERIALS AND METHODS

### *In silico* genomic database analysis and secondary structure prediction

We searched for candidates Nups using protein BLAST on the NCBI website and *Tetrahymena* Genome Database Wiki (http://ciliate.org/index.php/home/welcome) (Eisen et al., 2006; Stover et al., 2012). Expression profiles based on microarray data (http://tfgd.ihb.ac.cn/tool/exp) were obtained from the TetraFGD (http://tfgd.ihb.ac.cn/) (Miao et al., 2009). We identified the candidate proteins as Nups when the expression profile satisfied two conditions: first, that the amount of expression is lower in vegetative stages than in conjugation stages, and second, that expression peaks appear in between the C-2 and C-8 stages of conjugation. Secondary structures and transmembrane domains were predicted by PSIPRED (http://bioinf.cs.ucl.ac.uk/psipred/) and the TMHMM server (http://www.cbs.dtu.dk/services/TMHMM-2.0/), respectively. Coiled-coil regions were predicted through PBIL Coiled-Coils prediction (https://npsa-prabi.ibcp.fr/cgi-bin/npsa_automat.pl?page=npsa_lupas.html) or SIB COILS (http://embnet.vital-it.ch/software/COILS_form.html) tools. Conserved domains were searched for by using InterPro (http://www.ebi.ac.uk/interpro/).

### DNA construction

cDNAs were amplified by PrimeSTAR reagent (Takara, Kyoto, Japan) from the reverse transcripts prepared from the total RNA fraction of vegetative or conjugating cells as described previously (Iwamoto et al., 2009). The cDNAs were digested with *Xho*I and *Apa*I, and cloned into the pIGF1 vector to ectopically express them as N-terminal GFP-tagged proteins (Malone et al., 2005). The pIGF1C vector with the multi-cloning site at the 5′ site of the GFP-coding sequence was generated by modifying the pIGF1 vector, and used to ectopically express GFP-tagged Nup58 and Pom121 as C-terminal GFP-tagged proteins; the cDNAs of these Nups were cloned into the pIGF1C vector using the *Xho*I and *Kpn*I sites. To endogenously express Nups tagged with a fluorescent protein at the C-termini of the macronuclear ORFs, MicNup214, Nup160, and Nup133 were tagged with GFP using a pEGFP-neo4 vector (Mochizuki, 2008) (a kind gift from Kazufumi Mochizuki, Institute of Molecular Biotechnology of the Austrian Academy of Sciences, Vienna, Austria), MicNup153 was tagged with mNeon using a p2xmNeon_6xmyc_Neo4 vector (a kind gift from Aaron Turkewitz, University of Chicago, Chicago, IL), and Seh1 was tagged with mCherry using a pmCherry-pur4 vector (Iwamoto et al., 2014). Primers used in this study are listed in Table S7.

### Expression of GFP-tagged Nups in *Tetrahymena* cells

Conjugating cells were subjected to transfection by electroporation using a Gene Pulser II (Bio-Rad, Hercules, CA) as described previously (Iwamoto et al., 2014, 2015). The resulting cell suspension was cultivated for 18 h and then treated with paromomycin sulfate (Sigma-Aldrich, St Louis, MO) at 120 µg/ml when using pIGF1, pIGF1C, pEGFP-neo4 and p2xmNeon_6xmyc_Neo4 vectors, or puromycin dihydrochloride (Fermentek, Jerusalem, Israel) at 200 µg/ml when using a pmCherry-pur4 vector. Cadmium chloride was also added at 0.5 µg/ml to induce the expression of drug-resistant genes for pEGFP-neo4, p2xmNeon_6xmyc_Neo4, and pmCherry-pur4 vectors. Resistant cells usually appeared within a few days after the drug was added. We checked that at least five independent clones (i.e. grown in five different wells) exhibited the same intracellular localization of each GFP–Nup.

### Immunoprecipitation

For immunoprecipitation, GFP–Nup-expressing cells in logarithmic growth were pretreated with 0.5 mM phenylmethylsulfonyl fluoride (PMSF) for 30 min at 30°C and then collected by centrifugation (700 ***g*** for 1 min). The cells were resuspended at 2.5×10^6^ cells/ml in homogenization buffer composed of 150 mM NaCl, 1% Triton X-100, 2 mM PMSF, and Complete protease inhibitor cocktail (Roche Diagnostics, Mannheim, Germany), and then homogenized with sonication on ice. The supernatant obtained after centrifugation at 10,000 ***g*** for 15 min was pretreated with Protein-A–Sepharose to absorb non-specifically bound proteins. After removal of the beads by low-speed centrifugation (720 ***g*** for 5 min), the supernatant was incubated with 50 µg anti-GFP rabbit polyclonal antibody (#600-401-215, Rockland Immunochemicals, Limerick, PA) for 2 h at 4°C. To collect immunoprecipitated target proteins of interest, fresh Protein-A–Sepharose was added, incubated for another 2 h at 4°C, and then collected by centrifugation (720 ***g*** for 5 min). After a brief washing with homogenization buffer, the Sepharose beads were incubated with NuPAGE sample buffer (Thermo Fisher Scientific, Waltham, MA) to elute bound proteins. The proteins were separated by SDS-PAGE.

### Mass spectrometry analysis

The gel sample lane was cut into several pieces, and each treated with trypsin. The trypsinized peptide sample was subjected to liquid chromatography–tandem mass spectrometry (LC-MS/MS) using the LXQ linear ion trap (Thermo Finnigan, San Jose, CA) equipped with a Magic2002 and nanospray electrospray ionization device (Michrom BioResources, Auburn, CA and AMR, Tokyo, Japan), as described previously (Obuse et al., 2004). The LC-MS/MS data were searched by Mascot (Matrix Science, London, UK) with a non-redundant *T. thermophila*-specific database (25,131 sequences) constructed from the nr NCBI database. The resulting files were loaded into Scaffold software (Proteome Software, Portland, OR) for comparing identified proteins between samples.

### Microscopic observation

Intracellular localizations of GFP-tagged Nups were observed by performing fluorescence microscopy (IX-70; Olympus, Tokyo, Japan). Images were taken using the DeltaVision microscope system (GE Healthcare, Issaquah, WA) with oil-immersion objective lens UApo40 (NA=1.35) (Olympus). Line profiles of fluorescence intensity were obtained with a measurement tool included in the DeltaVision system. Background fluorescence was measured from the cytoplasm as an averaged value of 5×5 pixels and was subtracted from the peak values of fluorescence on the NE.

### Indirect immunofluorescence staining

*Tetrahymena* cells expressing GFP-tagged Nups were first fixed with cold methanol for 20 min, and then additionally fixed with 4% formaldehyde in PBS for 20 min. After treatment with 1% bovine serum albumin (BSA), cells were treated with 5 µg/ml anti-GLFG monoclonal antibody 21A10 for 2–3 h (Iwamoto et al., 2013). After washing with PBS, cells were treated with Alexa Fluor 594-conjugated goat anti-mouse IgG at 1:1000 dilution for 1 h (Thermo Fisher Scientific). Images of 40 z-sections with a 0.2-μm interval were taken for cells by using the DeltaVision microscope system with an oil immersion objective lens PlanApoN60OSC (NA=1.4) (Olympus), and were processed by deconvolution using SoftWoRx software equipped with the microscope.

### Immuno-electron microscopy

*Tetrahymena* cells expressing GFP-tagged Nups were fixed with 4% formaldehyde for 30 min. After washing three times with PBS, they were permeabilized with 0.1% saponin for 15 min at room temperature. After treatment with 1% BSA, cells were incubated with anti-GFP polyclonal antibody (cat. no. 600-401-215; Rockland Immunochemicals) at 1:200 dilution for 2 h, washed three times with PBS, then incubated with FluoroNano gold-conjugated anti-rabbit Fab′ also conjugated to Alexa Fluor 594 (Nanoprobes, Yaphank, NY) at 1:400 dilution for 1 h. The immunolabeled cells were fixed with 2.5% (w/v) glutaraldehyde (Nacalai tesque, Kyoto, Japan) for 1 h. After washing with 50 mM HEPES (pH 5.8), they were incubated with silver enhancement reagent (Tange et al., 2016) for 7 min. The reaction was stopped by washing three times with distilled water. Then the cells were post-fixed with 1% OsO_4_ for 15 min, electron stained with 2% uranyl acetate for 1 h, dehydrated with sequentially increased concentrations of ethanol and embedded in epoxy resin (Epon812). The ultrathin sections sliced from the resin block were stained with 4% uranyl acetate for 15 min and lead citrate (Sigma-Aldrich) for 1 min, and observed with a transmission electron microscope JEM-1400 (JEOL, Tokyo, Japan) with an acceleration voltage of 80 kV.

## References

[JCS199398C1] Alber F., Dokudovskaya S., Veenhoff L. M., Zhang W., Kipper J., Devos D., Suprapto A., Karni-Schmidt O., Williams R., Chait B. T. et al. (2007). The molecular architecture of the nuclear pore complex. *Nature* 450, 695-701. 10.1038/nature06405 18046406

[JCS199398C2] Allen N. P. C., Huang L., Burlingame A. and Rexach M. (2001). Proteomic analysis of nucleoporin interacting proteins. *J. Biol. Chem.* 276, 29268-29274. 10.1074/jbc.M102629200 11387327

[JCS199398C3] Amlacher S., Sarges P., Flemming D., van Noort V., Kunze R., Devos D. P., Arumugam M., Bork P. and Hurt E. (2011). Insight into structure and assembly of the nuclear pore complex by utilizing the genome of a eukaryotic thermophile. *Cell* 146, 277-289. 10.1016/j.cell.2011.06.039 21784248

[JCS199398C4] Andersen K. R., Onischenko E., Tang J. H., Kumar P., Chen J. Z., Ulrich A., Liphardt J. T., Weis K. and Schwartz T. U. (2013). Scaffold nucleoporins Nup188 and Nup192 share structural and functional properties with nuclear transport receptors. *Elife* 2, e00745 10.7554/eLife.00745 23795296 PMC3679522

[JCS199398C5] Asakawa H., Yang H.-J., Yamamoto T. G., Ohtsuki C., Chikashige Y., Sakata-Sogawa K., Tokunaga M., Iwamoto M., Hiraoka Y. and Haraguchi T. (2014). Characterization of nuclear pore complex components in fission yeast *Schizosaccharomyces pombe*. *Nucleus* 5, 149-162. 10.4161/nucl.28487 24637836 PMC4049921

[JCS199398C6] Berke I. C., Boehmer T., Blobel G. and Schwartz T. U. (2004). Structural and functional analysis of Nup133 domains reveals modular building blocks of the nuclear pore complex. *J. Cell Biol.* 167, 591-597. 10.1083/jcb.200408109 15557116 PMC2172596

[JCS199398C7] Bilokapic S. and Schwartz T. U. (2012). Molecular basis for Nup37 and ELY5/ELYS recruitment to the nuclear pore complex. *Proc. Natl. Acad. Sci. USA* 109, 15241-15246. 10.1073/pnas.1205151109 22955883 PMC3458321

[JCS199398C8] Brohawn S. G., Leksa N. C., Spear E. D., Rajashankar K. R. and Schwartz T. U. (2008). Structural evidence for common ancestry of the nuclear pore complex and vesicle coats. *Science* 322, 1369-1373. 10.1126/science.1165886 18974315 PMC2680690

[JCS199398C9] Bui K. H., von Appen A., DiGuilio A. L., Ori A., Sparks L., Mackmull M.-T., Bock T., Hagen W., Andrés-Pons A., Glavy J. S. et al. (2013). Integrated structural analysis of the human nuclear pore complex scaffold. *Cell* 155, 1233-1243. 10.1016/j.cell.2013.10.055 24315095

[JCS199398C10] Chug H., Trakhanov S., Hülsmann B. B., Pleiner T. and Görlich D. (2015). Crystal structure of the metazoan Nup62•Nup58•Nup54 nucleoporin complex. *Science* 350, 106-110. 10.1126/science.aac7420 26292704

[JCS199398C11] Cordes V. C., Reidenbach S., Rackwitz H.-R. and Franke W. W. (1997). Identification of protein p270/Tpr as a constitutive component of the nuclear pore complex-attached intranuclear filaments. *J. Cell Biol.* 136, 515-529. 10.1083/jcb.136.3.515 9024684 PMC2134304

[JCS199398C12] Cronshaw J. M., Krutchinsky A. N., Zhang W., Chait B. T. and Matunis M. J. (2002). Proteomic analysis of the mammalian nuclear pore complex. *J. Cell Biol.* 158, 915-927. 10.1083/jcb.200206106 12196509 PMC2173148

[JCS199398C13] DeGrasse J. A., DuBois K. N., Devos D., Siegel T. N., Sali A., Field M. C., Rout M. P. and Chait B. T. (2009). Evidence for a shared nuclear pore complex architecture that is conserved from the last common eukaryotic ancestor. *Mol. Cell. Proteomics* 8, 2119-2130. 10.1074/mcp.M900038-MCP200 19525551 PMC2742445

[JCS199398C14] Devos D., Dokudovskaya S., Alber F., Williams R., Chait B. T., Sali A. and Rout M. P. (2004). Components of coated vesicles and nuclear pore complexes share a common molecular architecture. *PLoS Biol.* 2, e380 10.1371/journal.pbio.0020380 15523559 PMC524472

[JCS199398C15] Eisen J. A., Coyne R. S., Wu M., Wu D., Thiagarajan M., Wortman J. R., Badger J. H., Ren Q., Amedeo P., Jones K. M. et al. (2006). Macronuclear genome sequence of the ciliate *Tetrahymena thermophila*, a model eukaryote. *PLoS Biol.* 4, e286 10.1371/journal.pbio.0040286 16933976 PMC1557398

[JCS199398C16] Enarson P., Enarson M., Bastos R. and Burke B. (1998). Amino-terminal sequences that direct nucleoporin nup153 to the inner surface of the nuclear envelope. *Chromosoma* 107, 228-236. 10.1007/s004120050301 9745047

[JCS199398C17] Fornerod M., van Deursen J., van Baal S., Reynolds A., Davis D., Murti K. G., Fransen J. and Grosveld G. (1997). The human homologue of yeast CRM1 is in a dynamic subcomplex with CAN/Nup214 and a novel nuclear pore component Nup88. *EMBO J.* 16, 807-816. 10.1093/emboj/16.4.807 9049309 PMC1169681

[JCS199398C18] Funakoshi T., Clever M., Watanebe A. and Imamoto N. (2011). Localization of Pom121 to the inner nuclear membrane is required for an early step of interphase nuclear pore complex assembly. *Mol. Biol. Cell* 22, 1058-1069. 10.1091/mbc.E10-07-0641 21289085 PMC3069009

[JCS199398C19] Goldfarb D. S. and Gorovsky M. A. (2009). Nuclear dimorphism: two peas in a pod. *Curr. Biol.* 19, R449-R452. 10.1016/j.cub.2009.04.023 19515351

[JCS199398C20] Gorlich D. and Mattaj I. W. (1996). Nucleocytoplasmic transport. *Science* 271, 1513-1518. 10.1126/science.271.5255.1513 8599106

[JCS199398C21] Grandi P., Doye V. and Hurt E. C. (1993). Purification of NSP1 reveals complex formation with “GLFG” nucleoporins and a novel nuclear pore protein NIC96. *EMBO J.* 12, 3061-3071. 7688296 10.1002/j.1460-2075.1993.tb05975.x PMC413571

[JCS199398C22] Grandi P., Dang T., Pané N., Shevchenko A., Mann M., Forbes D. and Hurt E. (1997). Nup93, a vertebrate homologue of yeast Nic96p, forms a complex with a novel 205-kDa protein and is required for correct nuclear pore assembly. *Mol. Biol. Cell* 8, 2017-2038. 10.1091/mbc.8.10.2017 9348540 PMC25664

[JCS199398C23] Greber U. F., Senior A. and Gerace L. (1990). A major glycoprotein of the nuclear pore complex is a membrane-spanning polypeptide with a large lumenal domain and a small cytoplasmic tail. *EMBO J.* 9, 1495-1502. 2184032 10.1002/j.1460-2075.1990.tb08267.x PMC551841

[JCS199398C24] Griffis E. R., Xu S. and Powers M. A. (2003). Nup98 localizes to both nuclear and cytoplasmic sides of the nuclear pore and binds to two distinct nucleoporin subcomplexes. *Mol. Biol. Cell* 14, 600-610. 10.1091/mbc.E02-09-0582 12589057 PMC149995

[JCS199398C25] Hallberg E., Wozniak R. W. and Blobel G. (1993). An integral membrane protein of the pore membrane domain of the nuclear envelope contains a nucleoporin-like region. *J. Cell Biol.* 122, 513-521. 10.1083/jcb.122.3.513 8335683 PMC2119659

[JCS199398C26] Hawryluk-Gara L. A., Shibuya E. K. and Wozniak R. W. (2005). Vertebrate Nup53 interacts with the nuclear lamina and is required for the assembly of a Nup93-containing complex. *Mol. Biol. Cell* 16, 2382-2394. 10.1091/mbc.E04-10-0857 15703211 PMC1087243

[JCS199398C27] Hodel A. E., Hodel M. R., Griffis E. R., Hennig K. A., Ratner G. A., Xu S. and Powers M. A. (2002). The three-dimensional structure of the autoproteolytic, nuclear pore-targeting domain of the human nucleoporin Nup98. *Mol. Cell* 10, 347-358. 10.1016/S1097-2765(02)00589-0 12191480

[JCS199398C28] Isgro T. A. and Schulten K. (2005). Binding dynamics of isolated nucleoporin repeat regions to importin-β. *Structure* 13, 1869-1879. 10.1016/j.str.2005.09.007 16338415

[JCS199398C29] Iwamoto M., Mori C., Kojidani T., Bunai F., Hori T., Fukagawa T., Hiraoka Y. and Haraguchi T. (2009). Two distinct repeat sequences of Nup98 nucleoporins characterize dual nuclei in the binucleated ciliate *Tetrahymena*. *Curr. Biol.* 19, 843-847. 10.1016/j.cub.2009.03.055 19375312

[JCS199398C30] Iwamoto M., Asakawa H., Hiraoka Y. and Haraguchi T. (2010). Nucleoporin Nup98: a gatekeeper in the eukaryotic kingdoms. *Genes Cells* 15, 661-669. 10.1111/j.1365-2443.2010.01415.x 20545767

[JCS199398C31] Iwamoto M., Asakawa H., Ohtsuki C., Osakada H., Koujin T., Hiraoka Y. and Haraguchi T. (2013). Monoclonal antibodies recognize gly-leu-phe-gly repeat of nucleoporin Nup98 of *Tetrahymena*, yeasts, and humans. *Monoclon. Antib. Immunodiagn. Immunother.* 32, 81-90. 10.1089/mab.2012.0118 23607342 PMC3732132

[JCS199398C32] Iwamoto M., Mori C., Hiraoka Y. and Haraguchi T. (2014). Puromycin resistance gene as an effective selection marker for ciliate *Tetrahymena*. *Gene* 534, 249-255. 10.1016/j.gene.2013.10.049 24185080

[JCS199398C33] Iwamoto M., Koujin T., Osakada H., Mori C., Kojidani T., Matsuda A., Asakawa H., Hiraoka Y. and Haraguchi T. (2015). Biased assembly of the nuclear pore complex is required for somatic and germline nuclear differentiation in *Tetrahymena*. *J. Cell Sci.* 128, 1812-1823. 10.1242/jcs.167353 25788697 PMC4432229

[JCS199398C34] Karrer K. M. (2012). Nuclear dualism. *Methods Cell Biol.* 109, 29-52. 10.1016/B978-0-12-385967-9.00003-7 22444142

[JCS199398C35] Keminer O. and Peters R. (1999). Permeability of single nuclear pores. *Biophys. J.* 77, 217-228. 10.1016/S0006-3495(99)76883-9 10388751 PMC1300323

[JCS199398C36] Kosova B., Panté N., Rollenhagen C. and Hurt E. (1999). Nup192p is a conserved nucleoporin with a preferential location at the inner site of the nuclear membrane. *J. Biol. Chem.* 274, 22646-22651. 10.1074/jbc.274.32.22646 10428845

[JCS199398C37] Liu S. M. and Stewart M. (2005). Structural basis for the high-affinity binding of nucleoporin Nup1p to the *Saccharomyces cerevisiae* importin-β homologue, Kap95p. *J. Mol. Biol.* 349, 515-525. 10.1016/j.jmb.2005.04.003 15878174

[JCS199398C38] Lutzmann M., Kunze R., Buerer A., Aebi U. and Hurt E. (2002). Modular self-assembly of a Y-shaped multiprotein complex from seven nucleoporins. *EMBO J.* 21, 387-397. 10.1093/emboj/21.3.387 11823431 PMC125826

[JCS199398C39] Loiodice I., Alves A., Rabut G., Van Overbeek M., Ellenberg J., Sibarita J. B. and Doye V. (2004). The entire Nup107-160 complex, including three new members, is targeted as one entity to kinetochores in mitosis. *Mol. Biol. Cell* 15, 3333-3344. 10.1091/mbc.E03-12-0878 15146057 PMC452587

[JCS199398C40] Malone C. D., Anderson A. M., Motl J. A., Rexer C. H. and Chalker D. L. (2005). Germ line transcripts are processed by a Dicer-like protein that is essential for developmentally programmed genome rearrangements of *Tetrahymena thermophila*. *Mol. Cell. Biol.* 25, 9151-9164. 10.1128/MCB.25.20.9151-9164.2005 16199890 PMC1265777

[JCS199398C41] Malone C. D., Falkowska K. A., Li A. Y., Galanti S. E., Kanuru R. C., LaMont E. G., Mazzarella K. C., Micev A. J., Osman M. M., Piotrowski N. K. et al. (2008). Nucleus-specific importin alpha proteins and nucleoporins regulate protein import and nuclear division in the binucleate *Tetrahymena thermophila*. *Eukaryot. Cell* 7, 1487-1499. 10.1128/EC.00193-08 18676955 PMC2547059

[JCS199398C42] Miao M., Ryan K. J. and Wente S. R. (2006). The integral membrane protein Pom34p functionally links nucleoporin subcomplexes. *Genetics* 172, 1441-1457. 10.1534/genetics.105.052068 16361228 PMC1456286

[JCS199398C43] Miao W., Xiong J., Bowen J., Wang W., Liu Y., Braguinets O., Grigull J., Pearlman R. E., Orias E. and Gorovsky M. A. (2009). Microarray analysis of gene expression during the *Tetrahymena thermophila* life cycle. *PLoS ONE* 4, e4429 10.1371/journal.pone.0004429 19204800 PMC2636879

[JCS199398C44] Mishra R. K., Chakraborty P., Arnaoutov A., Fontoura B. M. and Dasso M. (2010). The Nup107-160 complex and γ-TuRC regulate microtubule polymerization at kinetochores. *Nat. Cell Biol.* 12, 164-169. 10.1038/ncb2016 20081840 PMC2859955

[JCS199398C45] Mitchell J. M., Mansfeld J., Capitanio J., Kutay U. and Wozniak R. W. (2010). Pom121 links two essential subcomplexes of the nuclear pore complex core to the membrane. *J. Cell Biol.* 191, 505-521. 10.1083/jcb.201007098 20974814 PMC3003318

[JCS199398C46] Mochizuki K. (2008). High efficiency transformation of *Tetrahymena* using a codon-optimized neomycin resistance gene. *Gene* 425, 79-83. 10.1016/j.gene.2008.08.007 18775482

[JCS199398C47] Napetschnig J., Blobel G. and Hoelz A. (2007). Crystal structure of the N-terminal domain of the human protooncogene Nup214/CAN. *Proc. Natl. Acad. Sci. USA* 104, 1783-1788. 10.1073/pnas.0610828104 17264208 PMC1794303

[JCS199398C48] Obado S. O., Brillantes M., Uryu K., Zhang W., Ketaren N. E., Chait B. T., Field M. C. and Rout M. P. (2016). Interactome mapping reveals the evolutionary history of the nuclear pore complex. *PLoS Biol.* 14, e1002365 10.1371/journal.pbio.1002365 26891179 PMC4758718

[JCS199398C49] Obuse C., Iwasaki O., Kiyomitsu T., Goshima G., Toyoda Y. and Yanagida M. (2004). A conserved Mis12 centromere complex is linked to heterochromatic HP1 and outer kinetochore protein Zwint-1. *Nat. Cell Biol.* 6, 1135-1141. 10.1038/ncb1187 15502821

[JCS199398C50] Orias E. (2000). Toward sequencing the *Tetrahymena* genome: exploiting the gift of nuclear dimorphism. *J. Eukaryot. Microbiol.* 47, 328-333. 10.1111/j.1550-7408.2000.tb00057.x 11140445

[JCS199398C51] Orias E., Cervantes M. D. and Hamilton E. P. (2011). *Tetrahymena thermophila*, a unicellular eukaryote with separate germline and somatic genomes. *Res. Microbiol.* 162, 578-586. 10.1016/j.resmic.2011.05.001 21624459 PMC3132220

[JCS199398C52] Orjalo A. V., Arnaoutov A., Shen Z., Boyarchuk Y., Zeitlin S. G., Fontoura B., Briggs S., Dasso M. and Forbes D. J. (2006). The Nup107-160 nucleoporin complex is required for correct bipolar spindle assembly. *Mol. Biol. Cell* 17, 3806-3818. 10.1091/mbc.E05-11-1061 16807356 PMC1593160

[JCS199398C53] Osmani A. H., Davies J., Liu H.-L., Nile A. and Osmani S. A. (2006). Systematic deletion and mitotic localization of the nuclear pore complex proteins of *Aspergillus nidulans*. *Mol. Biol. Cell* 17, 4946-4961. 10.1091/mbc.E06-07-0657 16987955 PMC1679664

[JCS199398C54] Paine P. L., Moore L. C. and Horowitz S. B. (1975). Nuclear envelope permeability. *Nature* 254, 109-114. 10.1038/254109a0 1117994

[JCS199398C55] Rothballer A. and Kutay U. (2012). SnapShot: the nuclear envelope II. *Cell* 150, 1084-1084.e1. 10.1016/j.cell.2012.08.003 22939630

[JCS199398C56] Rout M. P., Aitchison J. D., Suprapto A., Hjertaas K., Zhao Y. and Chait B. T. (2000). The yeast nuclear pore complex: composition, architecture, and transport mechanism. *J. Cell Biol.* 148, 635-651. 10.1083/jcb.148.4.635 10684247 PMC2169373

[JCS199398C57] Siniossoglou S., Wimmer C., Rieger M., Doye V., Tekotte H., Weise C., Emig S., Segref A. and Hurt E. C. (1996). A novel complex of nucleoporins, which includes Sec13p and a Sec13p homolog, is essential for normal nuclear pores. *Cell* 84, 265-275. 10.1016/S0092-8674(00)80981-2 8565072

[JCS199398C58] Stavru F., Hülsmann B. B., Spang A., Hartmann E., Cordes V. C. and Görlich D. (2006). NDC1: a crucial membrane-integral nucleoporin of metazoan nuclear pore complexes. *J. Cell Biol.* 173, 509-519. 10.1083/jcb.200601001 16702233 PMC2063861

[JCS199398C59] Stover N. A., Punia R. S., Bowen M. S., Dolins S. B. and Clark T. G. (2012). *Tetrahymena* genome database Wiki: a community-maintained model organism database. *Database* 2012, bas007 10.1093/database/bas007 22434841 PMC3308163

[JCS199398C60] Strambio-de-Castillia C., Blobel G. and Rout M. P. (1999). Proteins connecting the nuclear pore complex with the nuclear interior. *J. Cell Biol.* 144, 839-855. 10.1083/jcb.144.5.839 10085285 PMC2148185

[JCS199398C61] Sugai T. and Hiwatashi K. (1974). Cytologic and autoradiographic studies of the micronucleus at meiotic prophase in *Tetrahymena pyriformis*. *J Protozool.* 21, 542-548. 10.1111/j.1550-7408.1974.tb03695.x 4214068

[JCS199398C62] Tamura K., Fukao Y., Iwamoto M., Haraguchi T. and Hara-Nishimura I. (2010). Identification and characterization of nuclear pore complex components in *Arabidopsis thaliana*. *Plant Cell* 22, 4084-4097. 10.1105/tpc.110.079947 21189294 PMC3027183

[JCS199398C63] Tange Y., Chikashige Y., Takahata S., Kawakami K., Higashi M., Mori C., Kojidani T., Hirano Y., Asakawa H., Murakami Y. et al. (2016). Inner nuclear membrane protein Lem2 augments heterochromatin formation in response to nutritional conditions. *Genes Cells* 21, 812-832. 10.1111/gtc.12385 27334362

[JCS199398C64] Terry L. J. and Wente S. R. (2009). Flexible gates: dynamic topologies and functions for FG nucleoporins in nucleocytoplasmic transport. *Eukaryot. Cell* 8, 1814-1827. 10.1128/EC.00225-09 19801417 PMC2794212

[JCS199398C65] Tetenbaum-Novatt J., Hough L. E., Mironska R., McKenney A. S. and Rout M. P. (2012). Nucleocytoplasmic transport: a role for nonspecific competition in karyopherin-nucleoporin interactions. *Mol. Cell. Proteomics* 11, 31-46. 10.1074/mcp.M111.013656 22357553 PMC3418842

[JCS199398C66] Vollmer B. and Antonin W. (2014). The diverse roles of the Nup93/Nic96 complex proteins - structural scaffolds of the nuclear pore complex with additional cellular functions. *Biol. Chem.* 395, 515-528. 10.1515/hsz-2013-0285 24572986

[JCS199398C67] Weirich C. S., Erzberger J. P., Berger J. M. and Weis K. (2004). The N-terminal domain of Nup159 forms a β-propeller that functions in mRNA export by tethering the helicase Dbp5 to the nuclear pore. *Mol. Cell* 16, 749-760. 10.1016/j.molcel.2004.10.032 15574330

[JCS199398C68] Winey M., Hoyt M. A., Chan C., Goetsch L., Botstein D. and Byers B. (1993). *NDC1*: a nuclear periphery component required for yeast spindle pole body duplication. *J. Cell Biol.* 122, 743-751. 10.1083/jcb.122.4.743 8349727 PMC2119589

[JCS199398C69] Wozniak R. W., Blobel G. and Rout M. P. (1994). POM152 is an integral protein of the pore membrane domain of the yeast nuclear envelope. *J. Cell Biol.* 125, 31-42. 10.1083/jcb.125.1.31 8138573 PMC2120016

[JCS199398C70] Yavuz S., Santarella-Mellwig R., Koch B., Jaedicke A., Mattaj I. W. and Antonin W. (2010). NLS-mediated NPC functions of the nucleoporin Pom121. *FEBS Lett.* 584, 3292-3298. 10.1016/j.febslet.2010.07.008 20624389

